# Transport properties through graphene with sequence of alternative magnetic barriers and wells in the presence of time-periodic scalar potential

**DOI:** 10.1038/s41598-021-92614-0

**Published:** 2021-06-24

**Authors:** Fatemeh Pakdel, Mohammad Ali Maleki

**Affiliations:** Department of Physics, University of Zanjan, University Blvd., 45371-38791 Zanjan, Iran

**Keywords:** Condensed-matter physics, Graphene

## Abstract

We investigate the electronic transport properties of a graphene sheet under the magnetic barriers and wells through the oscillating scalar potential combined with the static scalar potential barrier having two types of uniform and alternative profiles. We compute the total sideband transmission of the system by additional sidebands at energy, in presence of oscillating potential, $V_1$, using the transfer-matrix formalism and the Floquet sidebands series. The oscillating potential, generally, suppresses the Klein tunneling and the confinement of the charge carriers. In the absence of $V_1$, both profiles show the wave vector filtering effect for the carriers by controlling the energy *E* relative to the potential barrier height, $V_0$. The $(N-1)$-fold resonance splittings are observed through a region around $E=V_0$ with reduction of the transmission. The transmission vanishes in this region upon increasing the number of magnetic blocks *N*, strength of the magnetic field *B* in both configurations. We present an estimate relation for the width of the reduction region expressed in terms of *E*, $V_0$, *B* and the angle of incidence of the quasiparticles. We observe, in the second profile, $(N-1)$-fold resonances in the transmission for special values of $E=V_0$ with a separation depending on the width of the magnetic blocks. The magnetic field and the width of the magnetic blocks have critical values, where the transmission reduces to zero. All the features observed in the transmission reflect to the conductance. In both configurations, there are some peaks in the conductance corresponding to the resonances of the transmission. The oscillations of the conductance are obtained which was observed in the experimental results. We, also, find the possibility for switching the transport properties of the system by changing the characteristic parameters of the magnetic system.

## Introduction

The electronic transport properties of graphene subjected to inhomogeneous external scalar and vector potentials has opened a new era in graphene-based investigations^1–5^. The charge carriers in graphene with a linear dispersion near the Fermi points behave as massless fermions called as Dirac-Weyl (DW) quasiparticles^6^. Because of their different chirality, the Klein tunneling occures^1,7–9^. It causes perfect transmission and high mobility of graphene, but it sometimes makes the electrostatic confinement of the Dirac fermions difficult. The inhomogeneous magnetic field in graphene system can confine the charge carriers and control the transport properties of the system^3,6,10–13^. The combined effect of applied electrostatic potential and magnetic field induces interesting results upon transmission in the graphene junctions, such as Fabry-P$\acute{e}$rot interference^14–16^, the collapse of Landau levels^17^ and other new transport phenomena^18–20^. Recently, the study of periodically driven quantum systems^21^ has found particular importance for devices and optical applications as, experimentally, the time dependent effects^22–24^ and periodically driven tunnelling^25–29^.

Some theoretical literatures has studied the transport properties of monolayer graphene under influence of magnetic barriers and wells like wave vector filtering^3,6,30–33^ and resonance splitting effect through magnetic superlattices in absence of scalar potential^34^. The Klein tunneling in graphene heterojunctions has been investigated under the influence of perpendicular magnetic field via the non-equilibrium Green’s function method^16^. It has been found that in pnp graphene junctions with the perpendicular magnetic field, the strength of the magnetic field has a critical value in which the conductance vanishes. Also, a constriction region with low transmission is induced by potential barrier when the static scalar potential is close to the Fermi energy, so called the equal-barrier case. Harmonically driven scalar potential has been investigated in the monolayer graphene^35^. The perfect normal transmission which was observed in the static barrier case persists for the oscillating scalar potential barrier. Over the last decade, many theoretical works have been concentrated on the graphene sheet subjected to a magnetic field and an external time harmonic scalar potential^36,37^. Recently, through an experimental work, it has been reported that a magnetic field can control the negative differential conductance in scanning tunneling spectroscopy of graphene npn junction resonators^38^.

Motivated by the above issues, here, we focus on multiple magnetic barriers and wells (magnetic blocks) through the scalar square potential barrier harmonically oscillating in time as two types of one-dimensional profiles. A uniform scalar potential barrier is considered throughout the magnetic blocks in the former configuration while multiple scalar potential barriers alternatingly fit the magnetic barriers are considered in the latter profile. In order to calculate the total transmission probability, the Floquet sidebands series are used due to the numerical difficulties^25,26^. The external oscillating scalar potential is found, in both profiles, to act as a switch for the transport properties in the studied magnetic systems. The transmission and conductance are turned on or off by tuning the number of the magnetic blocks, strength of the magnetic field and width of the magnetic blocks. The wave vector filtering effect is observed in both profiles while, in the former configuration, the resonance effects are observed and a perfect transmission exists over a wide angular range. The Klein tunneling, in both configurations, is suppressed for magnetic fields weaker than a critical value and the conductance drops to zero. For weak magnetic fields, the transmission and the conductance are reduced by increasing width of the magnetic blocks and are suppressed for larger thicknesses. The $(N-1)$-fold resonance splitting peaks are observed for the magnetic system with *N* magnetic blocks. Moreover, in the latter profile, these resonance peaks are seen for special values of the energy in the equal-barrier case. The wave vector filtering limitation is suppressed by applying the oscillating scalar potential and the resonances are replaced by peaks. The transmission and conductance, in the equal-barrier case, are increased by applying the oscillating scalar potential and reduced by increasing strength of the magnetic field and number of the blocks. The $(N-1)$-fold resonances are appeared by changing the magnetic field and are, also, observed in the incident angular distribution range.

The outline of the paper is organized as follows. In “Model and theoretical method section”, we introduce two profiles for the magnetic system and use the time-dependent DW Hamiltonian in order to calculate the transmission probability and the conductance. The Floquet theorem and transfer matrix technique are used in the framework of Landauer-Butticker formalisms. Our main physical results are, mainly, presented in “Results and discussion” section. Finally we draw the summary and conclusions in “Conclusion” section.

## Model and theoretical method

**Figure 1:**
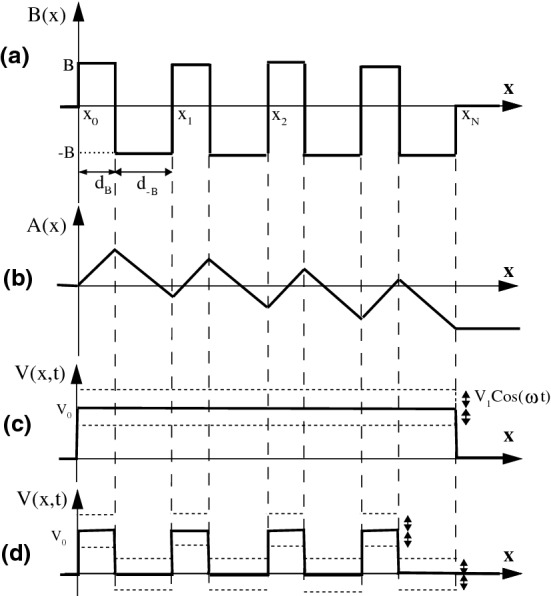
(**a**) The magnetic field profile consist of *N* magnetic barriers of width $d_B$ and height *B* separated by magnetic wells of width $d_{-B}$ and depth *B*. (**b**) The vector potential profile A(x) corresponding to *N* magnetic blocks of (**a**). (**c**) The uniform static scalar potential barrier profile of the first configuration with width $x_N$ and height $V_0$ that is oscillating sinusoidally with amplitude $V_1$ and frequency $\omega$. (**d**) The alternative static scalar potential barriers and wells, both oscillating sinusoidally with amplitude $V_1$ and frequency $\omega$, corresponding to the second profile.

We consider a monolayer graphene sheet in an external magnetic field subjected to a scalar potential energy. The magnetic field is along the *z*-direction as $\varvec{B}=B(x)\varvec{e}_z$ and has an alternating profile consisting of *N* magnetic barriers with heights *B* and widths $d_B$ and *N* magnetic wells with depth *B* and width $d_{-B}$ so that each barrier is followed by a well, as shown in Fig. 1a. The Landau gauge is chosen by considering $\varvec{A}=A(x)\varvec{e}_y$ with $B_z=\partial _xA(x)$, as illustrated in Fig. 1b. We introduce the magnitude of the vector potential, for our model, by

1 $$\begin{aligned} A(x)=\left\{ \begin{array}{ll} 0, &{} x\le 0; \\ (nD+x-x_n)B, &{} x_n\le x<x_n+d_B; \\ (nD+2d_B+x_n-x)B, &{}x_n+d_B\le x<x_{n+1}; \\ NDB, &{} x\ge x_N, \end{array}\right. \end{aligned}$$

where $x_n=n(d_B+d_{-B})$, running *n* from 0 to *N*. Here $D=d_B-d_{-B}$ and *DB* means the net magnetic flux through each block formed by a magnetic barrier and a magnetic well^6^. The total magnetic flux, *NDB*, is a controlling parameter for the transmission and the conductance. The scalar potential is zero in the non-magnetic regions while it oscillates in time around $V_0(x)$ with the frequency $\omega$ inside the magnetic region, as

2 $$\begin{aligned} V(x,t)=\left\{ \begin{array}{ll} V_0(x)+V_1\cos {\omega t}, &{} 0<x<x_N; \\ 0, &{} \hbox {otherwise}. \end{array}\right. \end{aligned}$$

We consider two types of the static scalar potential profiles, $V_0(x)$. The first profile, shown in Fig. 1c, consists of a uniform constant static scalar potential, given by

3 $$\begin{aligned} V_0(x)=V_0. \end{aligned}$$

It will be referred to this profile as the uniform static scalar potential and discussed in “Results and discussion” section. In the second configuration, illustrated in Fig. 1d, the static scalar potential changes, alternatively, from zero to $V_0$ as

4 $$\begin{aligned} V_0(x)=\left\{ \begin{array}{ll} V_0, &{} x_n\le x<x_n+d_B; \\ 0, &{}x_n+d_B\le x<x_{n+1}. \\ \end{array}\right. \end{aligned}$$

It will be referred to this profile as the alternative static scalar potential and discussed in “Conclusion” section.

The model described above can be realized if we first grow the monolayer graphene on a designed ferromagnetic sample and implant a top gate on it, then we apply the uniform or alternative voltage through the gate^37^. The two band DW Hamiltonian, including the magnetic field and the scalar potential energy is written as

5 $$\begin{aligned} H(x,t)=v_{\mathrm{F}}\varvec{\sigma }.(-i\hbar \varvec{\nabla } +e\varvec{A})+V(x,t){\mathbb {I}}_2, \end{aligned}$$

where $v_{\mathrm{F}}$ is the Fermi velocity ($\sim c/300$), $\varvec{\sigma }=(\sigma _x,\sigma _y)$ are the Pauli matrices^1^. Because of the conservation of the *y*-component of the momentum and the time periodicity of the Hamiltonian in Eq. (5), in accordance with the Floquet theory^25,39,40^, the full wave function can be written as

6 $$\begin{aligned} \Psi (x,y,t)=e^{ik_yy}e^{-iE_Ft/\hbar }f(t)\left( \begin{array}{c} \psi _1(x) \\ \psi _2(x) \\ \end{array}\right) , \end{aligned}$$

where $E_{\mathrm{F}}$ is the Floquet energy and *f*(*t*) is a periodic function with period $T=2\pi /\omega$. Here $\psi _1(x)$ and $\psi _2(x)$ are the spatial parts of the full wave function for each sub-lattice.

From now on, we express all the variables in dimensionless form using a typical strength of the magnetic field, $B_0$, and the magnetic length scale, $l_0=\sqrt{\hbar /(eB_0)}$. So, we represent all the lengths, time, $\omega$, $k_y$, *A*(*x*) and all the energy scales in the units of $l_0$, $l_0/v_{\mathrm{F}}$, $v_{\mathrm{F}}/l_0$, $l_0^{-1}$, $l_0B_0$ and $\hbar v_{\mathrm{F}}/l_0$, respectively. Using these considerations, the DW equation takes the form

7 $$\begin{aligned} \left( \begin{array}{cc} V(x,t) &{} -i\partial _x-i[k_y+A(x)] \\ -i\partial _x+i[k_y+A(x)] &{} V(x,t) \\ \end{array}\right) \Psi (x,y,t) =i\frac{\partial \Psi (x,t)}{\partial t}. \end{aligned}$$

In the magnetic region, inserting Eqs. (2) and (6) in Eq. (7), the following two coupled differential equations

8a $$\begin{aligned}&-i\left[ \frac{d}{dx}+(k_y+A(x))\right] \psi _2(x)= \left[ E_F-V_0(x)-V_1\cos ({\omega t}) +\frac{i}{f(t)}\frac{df(t)}{dt}\right] \psi _1(x), \end{aligned}$$

8b $$\begin{aligned}&-i\left[ \frac{d}{dx}-(k_y+A(x))\right] \psi _1(x)= \left[ E_F-V_0(x)-V_1\cos ({\omega t}) +\frac{i}{f(t)}\frac{df(t)}{dt}\right] \psi _2(x), \end{aligned}$$

are obtained for $\psi _1(x)$ and $\psi _2(x)$. The variables can be separated by introducing the Floquet sideband energy as

9 $$\begin{aligned} E_m=E_{\mathrm{F}}-V_1\cos {\omega t}+\frac{i}{f(t)}\frac{df(t)}{dt}, \end{aligned}$$

where *m* is an integer called the Floquet sideband index in the magnetic region^26,37^. So, the time dependent part of the wave function is obtained by solving Eq. (9) to get $f(t)\propto \exp {[-i(E_m-E_{\mathrm{F}})t-i(V_1/\omega )\sin {(\omega t)}]}$. The Jacob-Anger identity, $\exp (iz\sin \theta ) =\sum _{n=-\infty }^{\infty }J_n(z)\exp (in\theta )$, and periodicity of *f*(*t*) yield $E_m=E_{\mathrm{F}}+m\omega$. Then, the time dependent part is written as

10 $$\begin{aligned} f(t)=f_0\sum _{l,m=-\infty }^{\infty }J_{l-m}(V_1/\omega )e^{-il\omega t}, \end{aligned}$$

and the wave function in the magnetic region is

11 $$\begin{aligned} \Psi _{\pm B}(x,y,t)=e^{ik_yy}\sum _{l,m=-\infty }^{\infty }J_{l-m} \left( \frac{V_1}{\omega }\right) e^{-i(E_{\mathrm{F}}+l\omega )t} \left( \begin{array}{c} \psi _1^m(x) \\ \psi _2^m(x) \\ \end{array}\right) . \end{aligned}$$

The coupled Eqs. (8) can be rewritten, using Eq. (9), to get the following uncoupled differential equation

12 $$\begin{aligned} \frac{d^2\psi _1^m(x)}{dx^2}-\left[ (k_y+A(x))^2-(E_m-V_0(x))^2 +\frac{\partial A(x)}{\partial x}\right] \psi _1^m(x) =0. \end{aligned}$$

for the spinor $\psi _1^m(x)$. Replacing the variable *x* with $q=\sqrt{2/B}[k_y+A(x)]$, Eq. (12) takes the form

13 $$\begin{aligned} \frac{d^2\psi _1^m(q)}{dq^2}-\left( \frac{q^2}{4}+a_m\right) \psi _1^m(q)=0, \end{aligned}$$

which has two independent solutions $D_{-a_m-1/2}(q)$ and $D_{-a_m-1/2}(-q)$, known as the Parabolic Cylinder functions^41^. Here $a_m=-(E_m-V_0(x))^2/(2B)\pm 1/2$, where the upper (lower) sign corresponds to the magnetic barrier (well) region. So, the spinors $\psi _1^m(x)$ and $\psi _2^m(x)$ takes the forms

14a $$\begin{aligned}&\psi _1^m(x)=b_1^mD_{-a_m-1/2}(q)+b_2^mD_{-a_m-1/2}(-q), \end{aligned}$$

14b $$\begin{aligned}&\psi _2^m(x) =\frac{i\sqrt{2B}}{E_m-V_0}[b_1^mD_{-a_m+1/2}(q)-b_2^mD_{-a_m+1/2}(-q)], \end{aligned}$$

in the barriers, while for the wells they are

15a $$\begin{aligned}&\psi _1^m(x)=b_3^mD_{-a_m+1/2}(-q)+b_4^mD_{-a_m+1/2}(q), \end{aligned}$$

15b $$\begin{aligned}&\psi _2^m(x)=\frac{-i\sqrt{2B}}{E_m-V_0(x)}(-a_m+1/2) \times [b_3^mD_{-a_m-1/2}(-q)-b_4^mD_{-a_m-1/2}(q)], \end{aligned}$$

where $b_1^m$, $b_2^m$, $b_3^m$ and $b_4^m$ are constant coefficients.

In the non-magnetic region, solution of the matrix Eq. (7), for $x<0$, is

16 $$\begin{aligned} \Psi _0(x,y,t)=e^{ik_yy}\sum _{l,m=-\infty }^{\infty }\delta _{m,l}e^{-iE_lt} \left[ a_1^le^{ik_lx}\left( \begin{array}{c} 1 \\ \frac{k_l+ik_y}{E_l} \\ \end{array}\right) +a_2^le^{-ik_lx}\left( \begin{array}{c} 1\\ \frac{-k_l+ik_y}{E_l} \\ \end{array}\right) \right] , \end{aligned}$$

and

17 $$\begin{aligned} \Psi _N(x,y,t)=e^{ik_yy}\sum _{l,m=-\infty }^{\infty }\delta _{m,l}e^{-iE_lt} \left[ c_1^le^{ik_lx}\left( \begin{array}{c} 1 \\ \frac{k_l+i[k_y+A(x)]}{E_l} \\ \end{array}\right) +c_2^le^{-ik_lx}\left( \begin{array}{c} 1\\ \frac{-k_l+i[k_y+A(x)]}{E_l} \\ \end{array}\right) \right] , \end{aligned}$$

for $x>x_N$, where $a_1^l$ ,$a_2^l$ , $c_1^l$ and $c_2^l$ are constants and $k_l={\text {sgn}}(E_l)\sqrt{E_l^2-[k_y+A(x)]^2}$. The energy consists of *l* modes as $E_l=E_0+l\omega$, where $E_0$ is the lowest Floquet energy. For convenience we set $E_{\mathrm{F}}=E_0$.

Now we apply the boundary conditions at the boundaries $x=0, d_B, d_B+d_{-B} \cdots , x_N$, as

18 $$\begin{aligned} \Psi _0(0,y,t)=\Psi _{+B}(0,y,t), ~\Psi _{+B}(d_B,y,t)=\Psi _{-B}(d_B,y,t) \cdots , \Psi _{-B}(x_N,y,t)=\Psi _{N}(x_N,y,t). \end{aligned}$$

In the numerical calculation of the infinite summations in Eqs. (11), (16) and (17) the terms are terminated by running *l* and *m* from $-n$ to *n* where *n* is called the number of Floquet levels. In order to avoid the problem of divergency and get the accurate result, due to the oscillatory nature of the Bessel functions, it is necessary that the argument of the Bessel functions to be small, i.e. $n>V_1/\omega$. It means that the central time dependent potential region is weakly coupled to the other parts of the system. Using the orthogonality of the set of the oscillating functions $\exp {(il\omega t)}$, dropping the *t* and *y* parts, the wave functions can be written as

19a $$\begin{aligned}&\Psi _0(0)=W_0(0)\left( \begin{array}{c} a_1^l \\ a_2^l \\ \end{array}\right) , \end{aligned}$$

19b $$\begin{aligned}&\Psi _B(x)=W_B(x) \left( \begin{array}{c} b_1^m \\ b_2^m \\ \end{array}\right) , \end{aligned}$$

19c $$\begin{aligned}&\Psi _{-B}(x)=W_{-B}(x)\left( \begin{array}{c} b_3^m \\ b_4^m \\ \end{array}\right) , \end{aligned}$$

19d $$\begin{aligned}&\Psi _N(x_N)=W_0(x_N)W_N(x_N)\left( \begin{array}{c} c_1^l \\ c_2^l \\ \end{array}\right) , \end{aligned}$$

where

20a $$\begin{aligned}&W_0(x)=\left( \begin{array}{cc} {\mathbb {I}} &{} {\mathbb {I}} \\ {\mathbb {A}}_1^+(x) &{} {\mathbb {A}}_1^-(x) \\ \end{array}\right) , \end{aligned}$$

20b $$\begin{aligned}&W_N(x)=\left( \begin{array}{cc} {\mathbb {A}}_2^+(x) &{} {\mathbb {O}} \\ {\mathbb {O}} &{} {\mathbb {A}}_2^-(x) \\ \end{array}\right) , \end{aligned}$$

20c $$\begin{aligned}&W_{B}(x)=\left( \begin{array}{cc} {\mathbb {B}}_1^+ &{} {\mathbb {B}}_1^- \\ {\mathbb {B}}_2^+ &{} {\mathbb {B}}_2^- \\ \end{array}\right) , \end{aligned}$$

20d $$\begin{aligned}&W_{-B}(x)=\left( \begin{array}{cc} {\mathbb {C}}_1^+ &{} {\mathbb {C}}_1^- \\ {\mathbb {C}}_2^+ &{} {\mathbb {C}}_2^- \\ \end{array}\right) , \end{aligned}$$

with the corresponding matrix elements

21a $$\begin{aligned}&({\mathbb {A}}_1^{\pm }(x))_{lm}=\beta _l^{\pm }(x)\delta _{lm},\nonumber \\&({\mathbb {A}}_2^{\pm }(x))_{lm}=e^{\pm ik_lx}\delta _{lm}, \end{aligned}$$

21b $$\begin{aligned}&({\mathbb {B}}_1^{\pm })_{lm}=D_{-a_m-1/2}(\pm q)J_{l-m}(V_1/\omega )\nonumber \\&({\mathbb {B}}_2^{\pm })_{lm}=\pm \frac{i\sqrt{2B}}{E_m-V_0} D_{-a_m+1/2}(\pm q)J_{l-m}(V_1/\omega ), \end{aligned}$$

21c $$\begin{aligned}&({\mathbb {C}}_1^{\pm })_{lm}=D_{-a_m-1/2}(\mp q)J_{l-m}(V_1/\omega )\nonumber \\&({\mathbb {C}}_2^{\pm })_{lm}=\mp \frac{i\sqrt{2B}}{E_m-V_0(x)}(-a_m-1/2) D_{-a_m-3/2}(\mp q)J_{l-m}(V_1/\omega ), \end{aligned}$$

and

22 $$\begin{aligned} \beta _l^{\pm }(x)=\frac{\pm k_l+i[k_y+A(x)]}{E_l}. \end{aligned}$$

Then the transfer matrix defined by

23 $$\begin{aligned} \left( \begin{array}{c} a_1^l \\ a_2^l \\ \end{array}\right) =T \left( \begin{array}{c} c_1^l \\ c_2^l \\ \end{array}\right) , \end{aligned}$$

can be written as

24 $$\begin{aligned} T=W_0^{-1}(0)~W_{-B}(0)~T_0~ T_1\cdots T_{N-1}~ W_{-B}^{-1}(x_N)W_0(x_N)W_N(x_N), \end{aligned}$$

where

25 $$\begin{aligned} T_n=W_{-B}^{-1}(x_n)W_B(x_n)W_{B}^{-1}(x_n+d_B)W_{-B}(x_n+d_B). \end{aligned}$$

The notations ${\mathbb {I}}$ and ${\mathbb {O}}$ in Eqs. (20a) and (20b) indicate the unit and null matrices, respectively. The coefficients in Eqs. (16) and (17) are considered as $a_1^l=\delta _{l0}$ (because a single electron income from left side), $a_2^l=r_l$ (reflection amplitude), $c_1^l=t_l$ (transmission amplitude) and $c_2^l=0$.

The transfer matrix is written, in the matrix form, as

26 $$\begin{aligned} T=\left( \begin{array}{cc} {\mathbb {T}}_{11} &{} {\mathbb {T}}_{12} \\ {\mathbb {T}}_{21}&{}{\mathbb {T}}_{22}\\ \end{array}\right) , \end{aligned}$$

where ${\mathbb {T}}_{ij}$ are square matrices of order $2n+1$. Then

27 $$\begin{aligned} \left( \begin{array}{c} 0 \\ 0 \\ \cdot \\ \cdot \\ 1 \\ \cdot \\ \cdot \\ 0\\ 0 \\ \end{array}\right) ={\mathbb {T}}_{11}\left( \begin{array}{c} t_{-n} \\ t_{-n+1} \\ \cdot \\ \cdot \\ t_0 \\ \cdot \\ \cdot \\ t_{n-1} \\ t_{n} \\ \end{array}\right) . \end{aligned}$$

The transmission probability is obtained as

28 $$\begin{aligned} \tau _l(E,\varphi )=\frac{\cos \theta _l}{\cos \varphi }\vert t_l \vert ^2, \end{aligned}$$

where $\varphi$ is the incident angle measured with respect to the *x*-direction, $\theta _l=\tan ^{-1}[(k_y+A(x_N))/k_l(x_N)]$ and $t_l$ is defined through the Eq. (27). The factor $\cos \theta _l/\cos \varphi$ guaranties the flux conservation. Then, the total transmission probability from the set of barriers and wells is

29 $$\begin{aligned} \tau (E,\varphi )=\sum _{l=-n}^n\tau _l(E,\varphi ). \end{aligned}$$

Now, knowing the transmission, the zero-temperature conductance can be calculated through the Landauer-Buttiker formalism^42^

30 $$\begin{aligned} G(E)=G_0\int _{-\pi /2}^{\pi /2}\tau (E,\varphi )\cos {\varphi }d\varphi , \end{aligned}$$

with $G_0=2e^2EL_y/(\pi h)$ as the conductance unit where $L_y$ is the length of the graphene sample along the *y*-direction. Finally, we summarize the main parameters used in this section in Table 1.

**Table 1:** The main parameters used in “Model and theoretical method” section.

The parameter	Description	The parameter	Description
$\varvec{B}$	Magnetic field	$k_y$	*y*-component of the wave vector
$\varvec{A}$	Vector potential	$k_l$	Wave vector for *l*-th mode
*N*	Number of magnetic blocks	$\psi (x,y,t)$	Full wave function
$d_B$	Width of the barrier	$E_F$	Floquet energy
$d_{-B}$	Depth of the well	$E_0$	Lowest Floquet energy
$\varphi$	Incident angle	$E_m$	Floquet sideband energy
*V*(*x*, *t*)	Scalar potential	*m*	Floquet sideband index
$V_0$	Static scalar potential	*n*	Number of Floquet levels
$V_1$	Oscillating scalar potential	$\tau (E,\varphi )$	Total transmission probability
$\omega$	Angular frequency	*G*(*E*)	Conductance

## Results and discussion

In this section, the physical results of our numerical analysis on the transmission and conductance, given by Eqs. (29) and (30) are presented. The main interest is to analyse the effects of the incident angle, strength of the magnetic field, widths of the magnetic barriers and wells, energy, static and oscillating scalar potentials on the behavior of the transmission and conductance through the system shown in Fig. 1. The numerical results are generalized to the former and latter profiles in “Uniform static scalar potential” section and “Alternative static scalar potential” section, respectively. The resonance and Klein tunneling effects are studied in “Resonance and Klein” tunneling section and the conductance is investigated in “Conductance” section, for both configurations. The terms of the series in Eq. (29) are kept up to $n=5$. It has been considered $B_0=0.1$ T for the scaling value of the magnetic field. In this case, the length, angular frequency and energy scales are obtained to be $l_0=81.13$ nm, $v_{\mathrm{F}}/l_0=12.325\times 10^{12}$ rad/s and $\hbar v_{\mathrm{F}}/l_0=8.113$ meV, respectively. All of the coming figures are color online.

### Uniform static scalar potential

**Figure 2:**
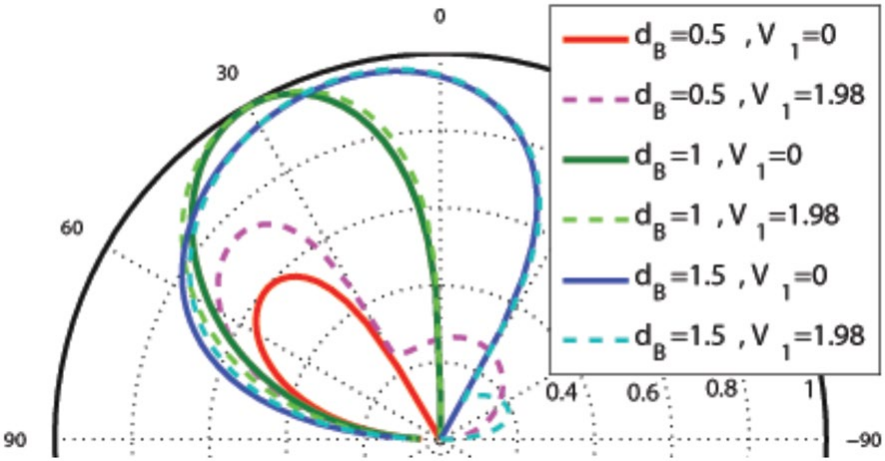
Angular dependence of the total sideband transmission for one magnetic block ($N=1$), $\omega =2$, $B=1$, $E=1$, $d_{-B}=2$ and $V_0=12$ for different values of $d_B$ and $V_1$.

In this subsection, we concentrate on the graphene-based system with the magnetic profile given by Eq. (1) and the scalar potential profile described by Eqs. (2) and (3), shown in Fig. 1a–c. In Fig. 2, the incident angle dependence of the transmission probability is depicted for different values of $d_B$, non-zero magnetic flux ($d_B\ne d_{-B}$) and $V_1=0, 1.98$. The used parameters are $B=1$, $E=1$, $d_{-B}=2$ and $V_0=12$ in the case of one magnetic block, i.e. $N=1$. There is an angular confinement for the quasiparticles in absence of the oscillating scalar potential, i.e. $V_1=0$. In the case of $d_B<d_{-B}$, it is specified by the critical value $\varphi _c=-\sin ^{-1}(1+(ND/E))$ for the incident angle^3,37^. The confinement is removed for non-zero values of $V_1$. This limitation can also be viewed for the energy of the quasiparticles as $E>\mid NBD/2\mid$. So, removing the oscillating scalar potential, turns off the transmission for $\varphi <\varphi _c$ or $E\le \mid NBD/2\mid$. Then, it is possible to confine the DW quasiparticles by turning off $V_1$ in the described magnetic structure. A perfect transmission is observed in $\theta =30^\circ$, for $d_B=1$, however the second lobe is very small. For the rest of this section, we concentrate on the case $d_B=d_{-B}$.

**Figure 3:**
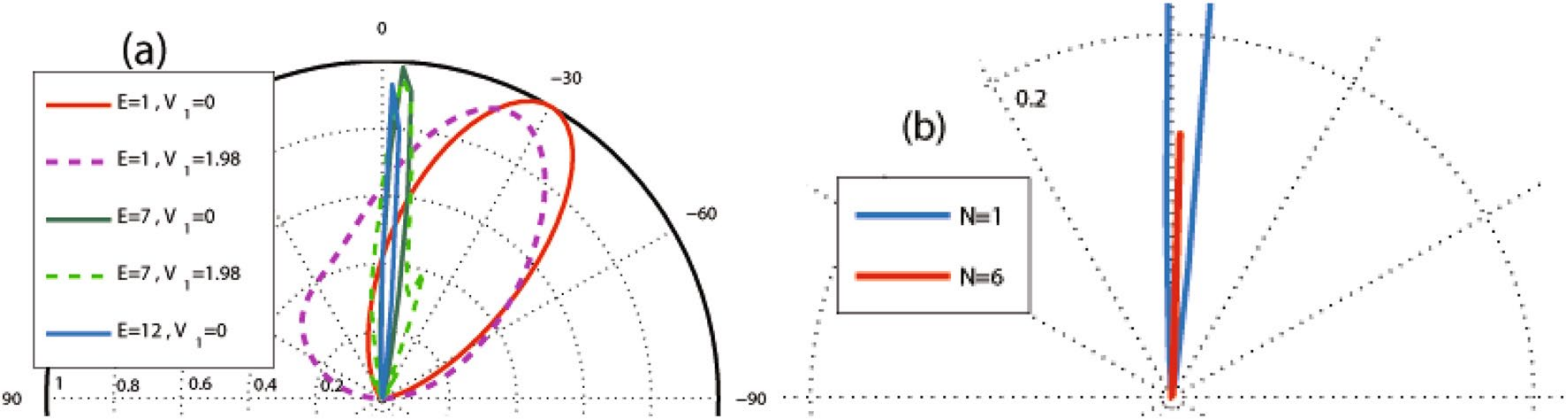
Angular dependence of the total sideband transmission for $\omega =2$, $B=1$, $d_B=d_{-B}=1$, (**a**) $N=1$ and different values of $V_0=E$ and $V_1$ and (**b**) $E=V_0=12$ and $V_1=0$ for $N=1, 6$.

**Figure 4:**
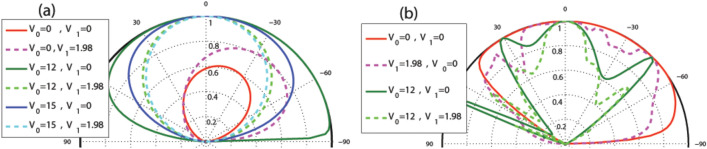
Angular dependence of the total sideband transmission for $N=1$ and (**a**) $E=1$ (**b**) $E=5$ varying $V_0$ and $V_1$. Other parameters are the same as in Fig. 3.

Figure 3a shows the angular dependence of transmission in the, so called, equal-barrier case, i.e. $E=V_0$, for one magnetic block with $B=1$ and $d_B=d_{-B}=1$. The curves are deflected towards negative angles. The transmission lobes become sharper by increasing the energy *E*. In Fig. 3b, it has been zoomed on the angular dependence of the transmission in the case of $E=V_0=12$ and $V_1=0$ for $N=1, 6$. The increment of the number of magnetic blocks makes the beams shorter and thinner. This induces a strong wave vector filtering and suppression in the transmission for the structure.

Figure 4a depicts that for $E=1$ and in the absence of the oscillating scalar potential, $V_1=0$, by applying the static potential $V_0$ the transmission increases and the Klein tunneling effect turns on. For the special value of $V_0=12$, a perfect transmission is observed in a wide range of the incident angle. In the absence of $V_0$, turning on $V_1$, the transmission increases. On the contrary, the transmission decreases when $V_1$ is turned on, in the presence of $V_0$. The Klein tunneling is observed for $V_0\ne 0$. For $E=5$, some changes appear in the mentioned behaviors (see Fig. 4b). In the absence of the scalar potential ($V_0=V_1=0$) there is a wide angular range with the perfect transmission. Applying $V_0$, $V_1$ or both of them, the transmission decreases over some parts of this range. The Klein tunneling is observed here, moreover, there are some angles indicating the resonance effects^6^.

**Figure 5:**
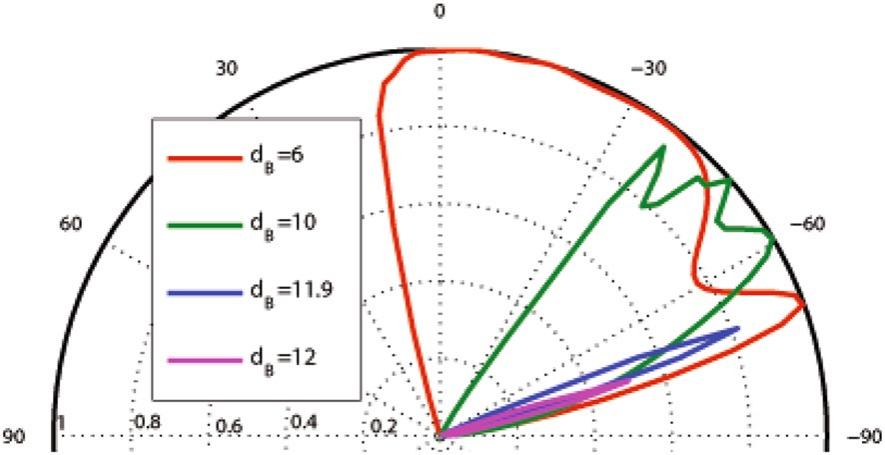
Angular dependence of the total sideband transmission for $N=1$ , $E=5$, $V_0=12$ and $V_1=0$ varying $d_B=d_{-B}$. Other parameters are the same as in Fig. 3.

**Figure 6:**
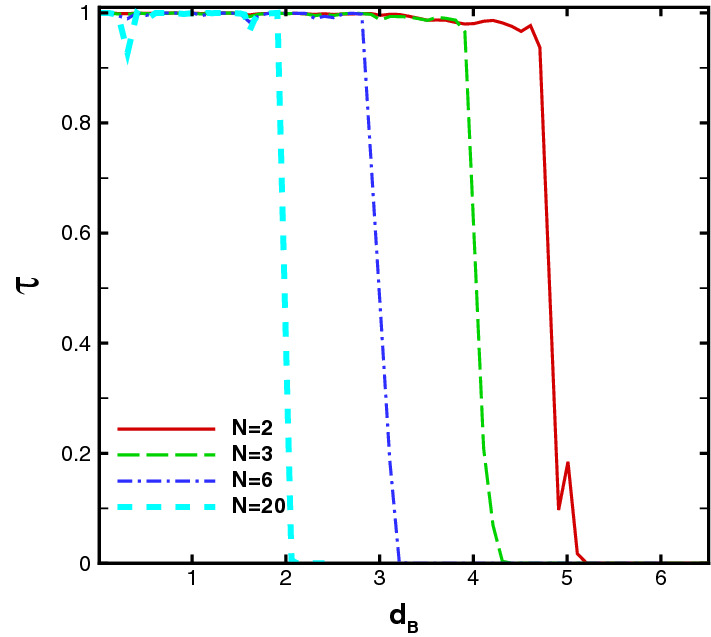
The total normal sideband transmission versus width of the magnetic barriers for $E=5$, $V_0=12$ and $V_1=1.98$ varying the number of the magnetic blocks. Other parameters are the same as in Fig. 3.

The width of the magnetic blocks is one of the important factors in the system which can affect the transmission and the Klein tunneling effects. Figure 5 shows the angular dependence of the transmission for $N=1$, $E=5$, $V_0=12$, $V_1=0$ and different values of $d_B$. Increasing $d_B$, the transmission pattern becomes narrower and shorter in the negative angle side. The beam disappears around a critical value given by $d_B=V_0/(ev_{\mathrm{F}}B)$ which, in the dimensionless notation, reads to $d_B=V_0/B$. For $V_0=0$, this critical width is given by $d_B=2E/(ev_{\mathrm{F}}B)$ which is the diameter of the cyclotron orbit or $d_B=2E/B$ in the dimensionless form. The critical value of $d_B$ decreases by turning $V_1$ on. In order to observe the response of the system to the Klein tunneling, we concentrate on the normal transmission. In Fig. 6 the normal transmission is plotted versus $d_B$ for $E=5$, $V_0=12$, $V_1=1.98$ and different values of *N*. For small values of $d_B$ the Klein tunneling is governed in the system, whereas for $d_B$ greater than a critical value the normal transmission falls to zero. This critical value of $d_B$ decreases by increasing the number of blocks.

### Alternative static scalar potential

**Figure 7:**
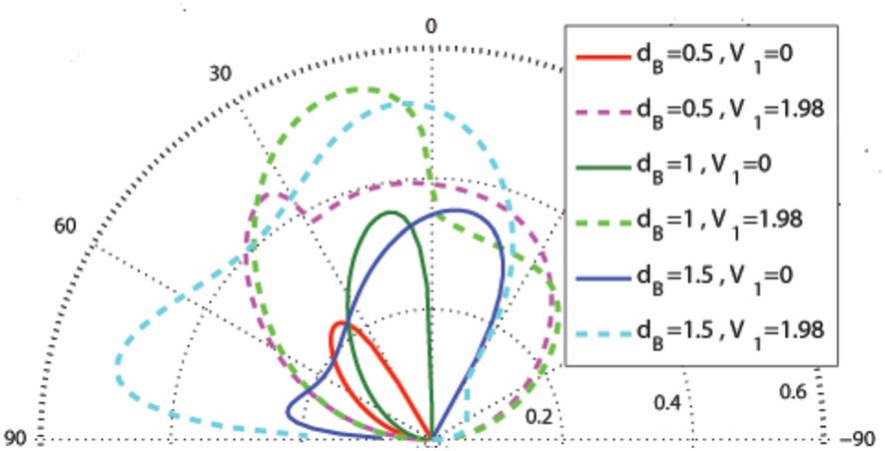
Angular dependence of the total sideband transmission for one magnetic block ($N=1$), $E=1$, $d_{-B}=2$ and $V_0=12$ for different values of $d_B$ and $V_1$. Other parameters are the same as in Fig. 3.

**Figure 8:**
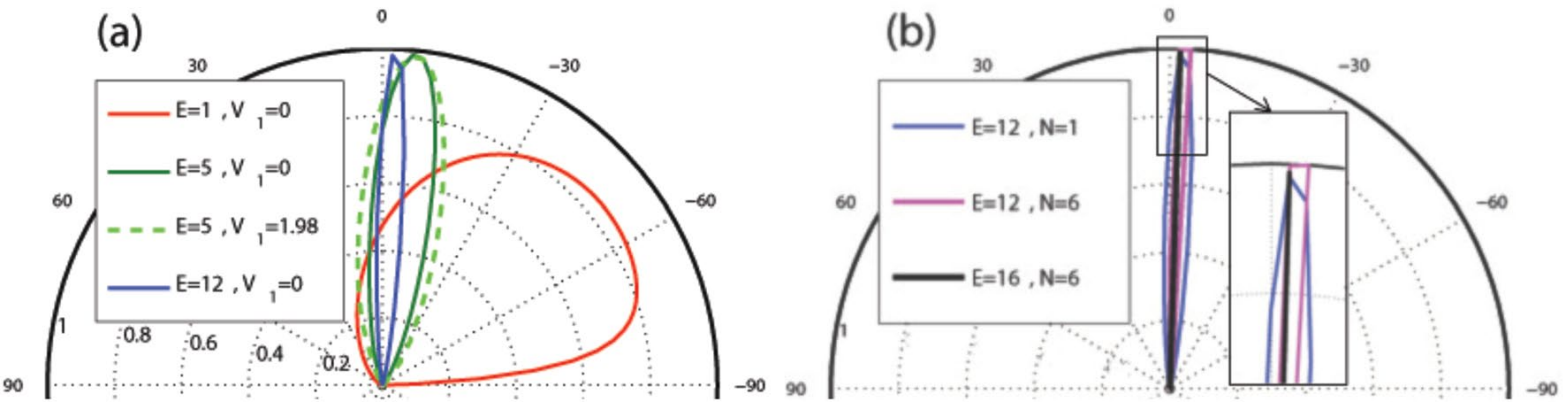
Angular dependence of the total sideband transmission for (**a**) $N=1$ and different values of $E=V_0$ and $V_1$, (**b**) $V_1=0$ and different values of $E=V_0$ and *N*. Other parameters are the same as in Fig. 3.

In this subsection, we consider the graphene-based system with the magnetic profile given by Eq. (1), similar to the case considered in “Results and discussion” section, while the scalar potential profile is described by Eqs. (2) and (4) which is shown in Fig. 1a, b and d. In Fig. 7, the effect of non-zero magnetic flux is shown on the incident angle dependence of the transmission probability for $V_1=0, 1.98$. The whole parameters are the same as in Fig. 2 in the previous configuration. The transmission is decreased in comparison to the uniform static scalar potential case. There is the same limitation for the incident angle as in the first profile, i.e. $\varphi >-\sin ^{-1}(1+(NBD/E))$ for $d_B<d_{-B}$, $\varphi <\sin ^{-1}(1-(NBD/E))$ for $d_B>d_{-B}$ and $E>\mid NBD/2\mid$ for the energy in absence of $V_1$, due to the conservation of $k_y$. Again, turning $V_1$ on, this confinement is removed. In contrary with the first configuration, the transmission increases by turning $V_1$ on. From now on, we consider $d_B=d_{-B}$.

Corresponding to Fig. 3 in the previous configuration, Fig. 8 is obtained for the angular dependence of transmission at $E=V_0$, in this profile. Here, also, increment of the energy makes the lobes sharper in negative angles and applying $V_1$ makes the lobes wider, which is apparent in Fig. 8a. The lobes become sharper for $N>1$, as it is seen in Fig. 8b. So, a strong wave vector filtering can be achieved by choosing the large values for the energy in the case of multiblock magnetic system.

**Figure 9:**
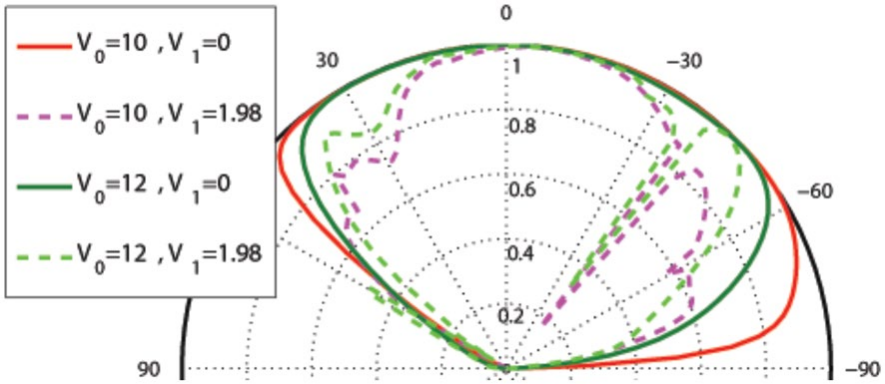
Angular dependence of the transmission for $N=1$, $E=5$ and different values of $V_0$ and $V_1$. Other parameters are the same as in Fig. 3.

**Figure 10:**
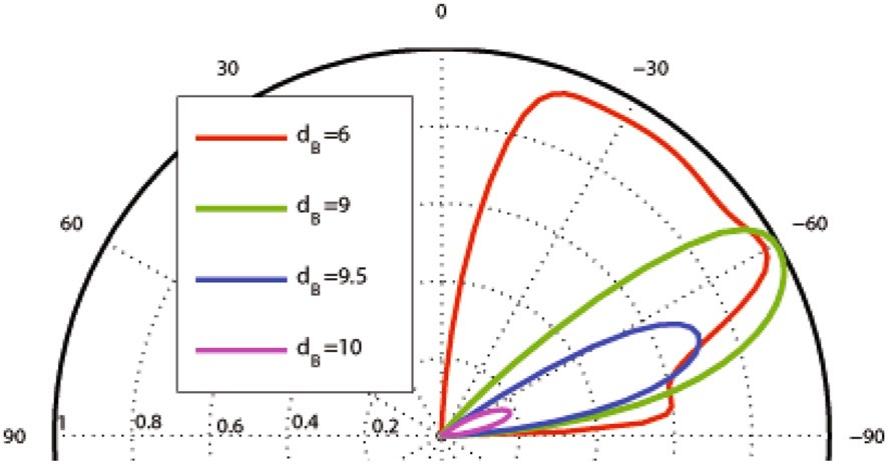
Angular dependence of the transmission for $N=1$, $E=5$, $V_0=12$ and $V_1=0$ varying $d_B=d_{-B}$. Other parameters are the same as in Fig. 3.

Now we analyze the angular dependence of the transmission with respect to the variation of $V_0$ and $V_1$. The same results shown before in Fig. 4 is, exactly, obtained for $V_0=0$. Again, there is a small range of the incident angles with low transmission for $E=1$ and a wide perfect transmission range for $E=5$ and $V_1=0$. In Fig. 9, the angular dependence of the transmission is depicted varying $V_0$ (non-zero) and $V_1$ for $E=5$. The Klein tunneling is observed for all values of $V_0$ and $V_1$. There is a wide angular range with perfect transmission for $V_1=0$. Turning $V_1$ on, the angular range of the transmission is not changed but some resonance effects are created in the edges of this range and the transmission is decreased. The same angular profile of the transmission is obtained for $V_0=0$ and $V_0=10$ in the absence of $V_1$, which can be justified by invariance of the cyclotron radius $r_c=\vert E-V_0\vert /ev_{\mathrm{F}}B$. Figure 10 shows the angular dependence of the transmission for different values of $d_B=d_{-B}$. Again, the transmission lobes are deflected towards negative angles. The transmission of the magnetic system decreases by increasing the thickness of the single magnetic block system in the absence of the oscillating scalar potential, $V_1$. The transmission lobes disappear at a critical value given by the cyclotron orbit diameter, $d_B=2E/B$.

### Resonance and Klein tunneling

**Figure 11:**
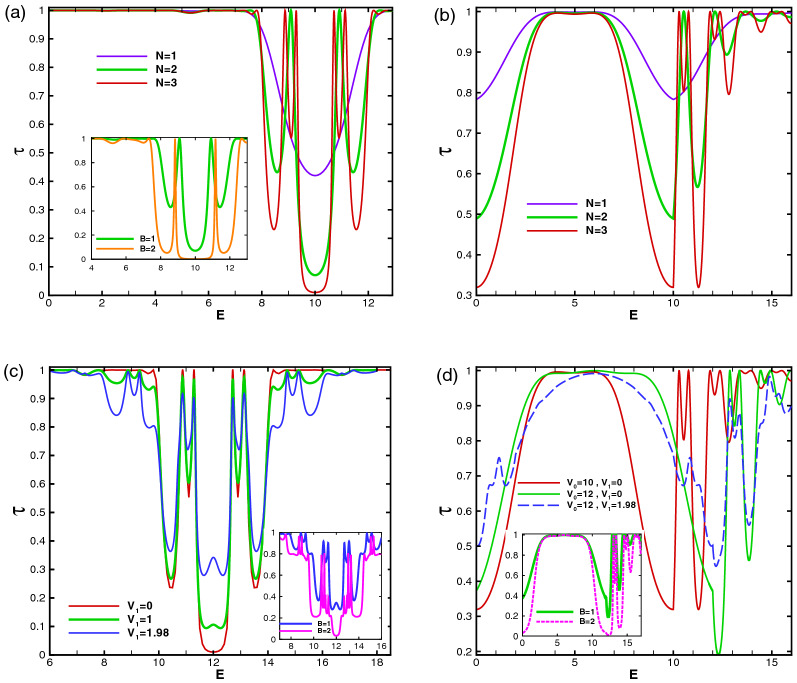
(**a**) The total normal sideband transmission as a function of the energy for the first configuration with $B=1$, $V_0=10$ and $V_1=0$ for different values of *N*. The inset is for $N=2$ and $B=1, 2$. (**b**) The normal transmission versus energy for the second profile with $B=1$, $V_0=10$ and $V_1=0$ for different values of *N*. (**c**) The normal transmission versus energy for the first configuration with $B=1$, $V_0=12$, $N=3$ and different values of $V_1$. The inset is for $V_1=1.98$ and $B=1, 2$. (**d**) The normal transmission versus energy for the second profile with $B=1$, $N=3$ and different values of $V_0$ and $V_1$. The inset is for $V_0=12$, $V_1=0$ and $B=1, 2$. Other parameters are the same as in Fig. 3.

In this subsection, we study the dependence of the total sideband transmission probability on the parameters of the system as the energy, the scalar potential and strength of the magnetic field. Our aim is investigation of resonances and Klein tunneling effect in the normal incidence for two profiles investigated in “Results and discussion” section and “Conclusion” section. In Fig. 11, the normal transmission is plotted versus the energy for the former (Fig. 11a and c) and latter (Fig. 11b and d) configurations, respectively. In both profiles, in the absence of $V_1$, there is a transmission drop region in the energy interval, given by $\vert E-V_0(x)\vert <\vert k_y+B\vert$, inside which the transmission experiences some resonance peaks^34^. This limitation can be expressed in terms of the energy, magnetic field or incident angle by sharing between this condition for different values of vector potential. This is due to the fact that the vector potential, *A*, takes values between zero and $Bd_B$, for $d_B=d_{-B}$. The eigenstates in the barriers are evanescent and they propagate in the well to form quasibound states, for $N\ge 2$. If the incident energy coincide with this bound state energies in the well, the transmission resonances occurs^43^. So, in this region, the transmission reduces except in resonance peaks. The degenerate eigenlevels in the wells split because of the coupling between the wells via tunneling in the barriers and it leads to the ($N-1$)-fold resonance splittings for *N* magnetic blocks (see Fig. 11a and b). Far from this region, the eigenstates are propagating states and the DW quasiparticles can transmit perfectly, so the transmission approaches to unity and the Klein tunneling is governed in the magnetic system. There are, also, $(N-1)$ distinct peaks, associated to the Fabry-P$\acute{e}$rot interference, because of interaction between the static and magnetic barriers and wells. The resonance splitting effect exists in the magnetic superlattice (the magnetic system with $N\ge 2$) versus energy, through an electrostatic barrier with the suppression of Klein tunneling. Applying $V_1$ reduces height of the resonance peaks from unity and by increasing $V_1$ other sets of $(N-1)$-fold resonances are appeared in the vicinity of $E=V_0$. The ($N-1$)-fold resonance peaks, also, are observed for $k_y\ne 0$.

In the first configuration (uniform static scalar potential) the curve of transmission versus energy exhibits a cusp at $E=V_0$ for $B=1$ and non-zero $V_1$. The cusp is removed for $V_1=0$ where the transmission is suppressed (see Fig. 11c). The cusps, induced by the oscillating potential, for more than one magnetic block grow up by the amplitude of the oscillating potential and the number of blocks. They are attributed to the Fabry-P$\acute{e}$rot fringes. The transmission around $E=V_0$ can be turned on (off), for multiple magnetic blocks with $N\ge 4$, just by turning on (off) $V_1$. The transmission in $E=V_0$ decreases by decreasing $V_1$ and it approaches zero for $V_1=1.98$ and large number of blocks, i.e. $N>40$. By increasing the difference between $V_0$ and *E*, the Klein tunneling appears and this occurs in higher differences by increasing $V_1$. Increasing *B* can also turn off the normal transmission (see the inset of Fig. 11a in the absence and the inset of Fig. 11c in the presence of $V_1$). Figure 11b and d show similar effects for the second profile (alternative static scalar potential). Here, unlike the first configuration, the energy *E* and the static scalar potential $V_0$ have not the same roles and the transmission shows completely non-symmetric behavior in two sides of $E=V_0$. In the absence of $V_1$, the transmission around $E=V_0$ reduces to zero by increasing the number of blocks ($N\ge 6$) or *B*. There are not any resonance peak for $E<V_0$. For $V_0\ge 5$, a Klein tunneling region is appeared, centered around $E=V_0/2$, which its width increases by increasing $V_0$. Here, the barrier and well modes are similar to each other due to the fact $\vert E-V_0\vert =E$ which was, also, seen in the first configuration. The transmission decreases from unity in two areas in both sides of this region, given by $\vert E-V_0(x)\vert <\vert k_y+A\vert$. Out of this range, for $E>V_0$, the Klein tunneling appears again and also alternative sets of $(N-1)$-fold resonances are observed. Turning $V_1$ on, the transmission around $E=V_0$ increases and the resonance peaks are changed to the usual peaks. The considerations studied here, can be used in designing switching on or off instruments for the transmission of the charge carriers in the graphene based systems.

**Figure 12:**
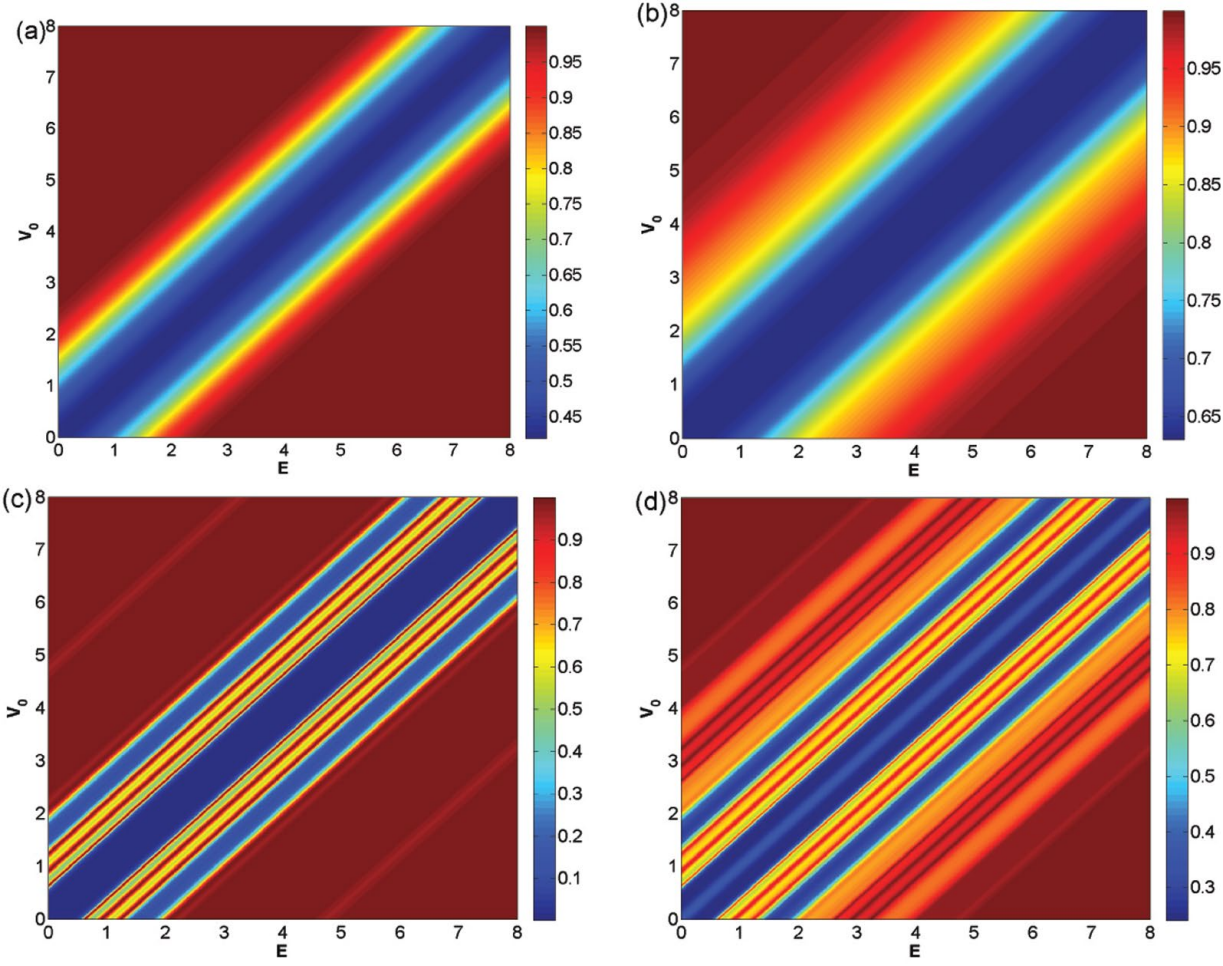
The total normal sideband transmission versus *E* and $V_0$ for the former profile, (**a**) $N=1$ and $V_1=0$, (**b**) $N=1$ and $V_1=1.98$, (**c**) $N=4$ and $V_1=0$ and (**d**) $N=4$ and $V_1=1.98$. Other parameters are the same as in Fig. 3.

The counter plots for the normal transmission for the first configuration are sketched in Fig. 12 versus the energy and the static scalar potential for $N=1, 4$ in both cases of absence and presence of the oscillating scalar potential. The energy *E* and the static scalar potential $V_0$ have similar roles in this configuration, which is not the case in second profile. In the single block case (Fig. 12a and b), there are ribbon bands in the main diameters, i.e. $E=V_0$, which indicates the non-zero minimum for the transmission which its width increases by increasing $V_1$. Moving away from the main diameter, the difference between *E* and $V_0$ increases and the normal transmission grows towards the unity which leads to the Klein tunneling effect. From the semiclassical point of view^44^, the Dirac fermions subjected to a perpendicular magnetic field are rotating around a circular orbit with the radius of cyclotron radius. Increasing the difference between energy and static scalar potential increases the cyclotron radius and the Dirac fermions can easily complete their cyclotron orbits, so the transmission and conductance are increased. In the case of four magnetic blocks (Fig. 12c and d) the 3-fold resonance lines are appeared close the main diameters. Here, the minimum transmission is zero for multiblock magnetic system, in the absence of $V_1$ (see Fig. 12c). So, the possibility for switching the total normal transmission to zero is provided by choosing $V_0$ and *E* close to each other for $N>3$ in the absence of $V_1$. The number of Fabry-P$\acute{e}$rot fringe patterns (resonances and peaks) increases in the presence of $V_1$ for the multiple magnetic blocks, symmetrically on both sides of the ribbon band. The line in the center of the main diameter in Fig. 12d corresponds to a cusp for $E=V_0$ in Fig. 11 which is created and grows by increasing *N* for $B=1$. The appearance of the second $(N-1)$-fold resonance lines are, also, observed in both sides of the main diameter, in Fig. 12d, by applying $V_1=1.98$.

**Figure 13:**
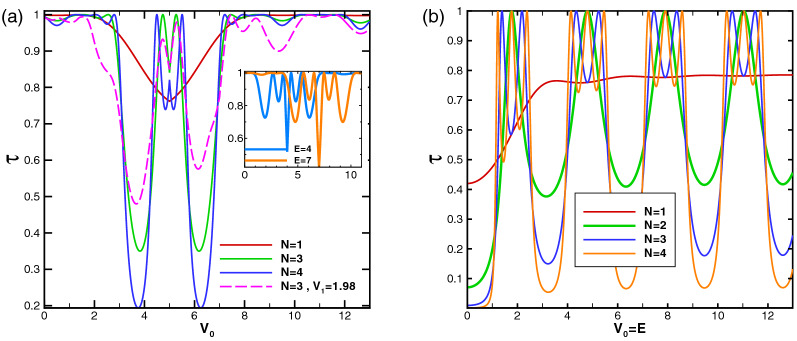
The total normal sideband transmission as a function of the static scalar potential for the latter configuration, (**a**) $E=5$ varying $N~,~V_1$. The inset is sketched for $N=3~,~V_1=0$ varying *E*, (**b**) the static scalar potential equals to the energy and $V_1=0$ varying *N*. Other parameters are the same as in Fig. 3.

In Fig. 13a dependence of the normal transmission to $V_0$ is investigated in the alternative static scalar potential case. The transmission drop region for $V_0$ is $E-\vert k_y+B\vert<V_0<E+\vert k_y+B\vert$ which consists of the fluctuations as $(N-1)$-fold resonances centered at $V_0=E$. In the presence of $V_1$ the resonance, asymmetrically, alters to the peaks and the fluctuations outside this region are exacerbated. For even number of blocks, the central peak does not include perfect transmission like the others. Increasing *N*, increases the amplitude of the transmission drop. These behaviors for the transmission are strongly affected by the energy. Depending on the value of the energy, it shows two types of behaviors. In Fig. 13a the energy was $E=5$ and, in its inset, the behavior of the transmission is shown for the energies $E=4, 7$ with $N=3$ and $V_1=0$. For these energies, two $(N-1)$-fold resonances are observed in both sides of $V_0=E$ and the transmission drops in $V_0=E$. In Fig. 13b the behavior of the transmission is studied versus $V_0=E$. The transmission, for one magnetic block, increases up to the energies around $V_0=E=3$ and, then, is going to be satisfied in $\tau =0.8$. For multiple blocks, the transmission takes oscillatory behavior due to the multireflections in the walls of the magnetic barriers. The period of this oscillations depends on $d_B=d_{-B}$. The $(N-1)$-fold resonances are, also, observed as before. So, unlike the first configuration, the transmission is not decreased for $V_0=E$ but the perfect transmissions are, also, observed in the resonance peaks.

**Figure 14:**
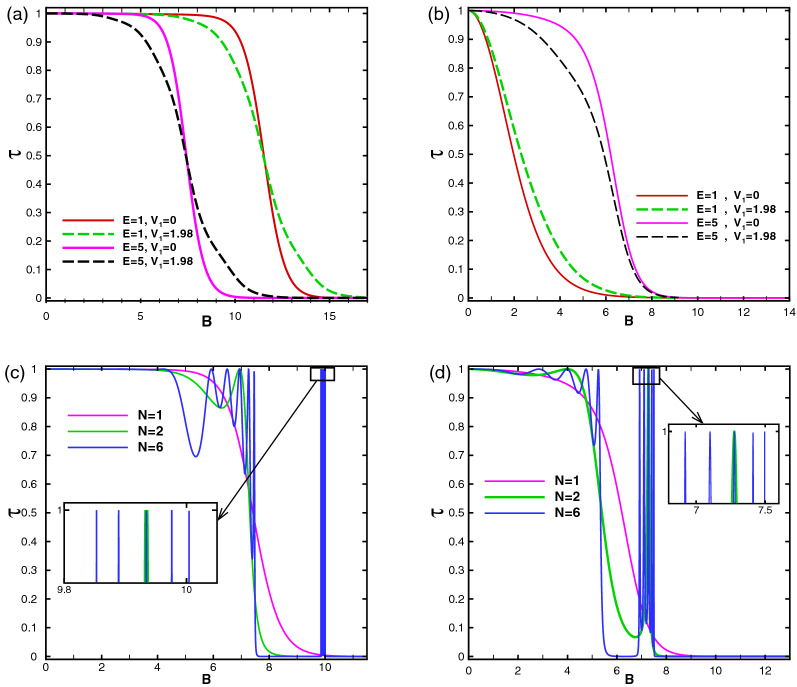
The total normal sideband transmission as a function of the strength of the magnetic field for (**a**) the former and (**b**) the latter profiles with $N=1$ and $V_0=12$ for different values of *E* and $V_1$ and (**c**) the former and (**d**) the latter configurations with $E=5$, $V_0=12$ and $V_1=0$ for different values of *N*. The insets zoom out the resonances. Other parameters are the same as in Fig. 3.

Figure 14 shows the total normal transmission of the Dirac fermions versus the magnetic field strength for different values of energy, oscillating scalar potential and number of magnetic blocks in the former and latter profiles. In the former configuration, as is shown in Fig. 14a, the Klein tunneling is observed for weak magnetic fields and it is suppressed in the first critical magnetic field with the magnitude $B_{c1}$. The transmission decreases and vanishes around a second critical field $B_{c2}$. The radius of the cyclotron orbit decreases with the increment of *B* and the transmission vanishes^16^. The critical fields $B_{c1}$ and $B_{c2}$ depend on *E*, $V_0$, $V_1$ and *N*. They both decrease with the increment of the energy, for $E<V_0$, due to the spreading of the scalar potential barrier in the whole magnetic region. Applying $V_1$, the first (second) critical field decreases (increases) and the difference $B_{c2}-B_{c1}$ increases. $B_{c2}$ decreases by increasing *N*, as is shown in Fig. 14c. The same analysis is performed for the latter configuration in Fig. 14b and d with the same parameters of the first profile. The first critical magnetic field is almost zero but the second is decreased, in comparison with the former configuration. $B_{c2}$ increases by the increment of the energy and is independent of $V_1$. In both profiles, by adding number of the magnetic blocks, two sets of $(N-1)$-fold resonances are appeared; the first between $B_{c1}$ and $B_{c2}$ and the second after $B_{c2}$. It has been observed that they are transformed, in the presence of $V_1$, to the fluctuations with non-perfect transmission. The perfect and zero transmission regions are separated from each other by a drop region which its width decreases by increment of *N*. For large number of magnetic blocks this region becomes narrower and the zero transmission region is obtained by the condition $\vert E-V(x)\vert <\vert k_y+B\vert$ which yields $B>7$ and $B>5$ in the first (Fig. 14c) and second (Fig. 14d) configurations, respectively.

**Figure 15:**
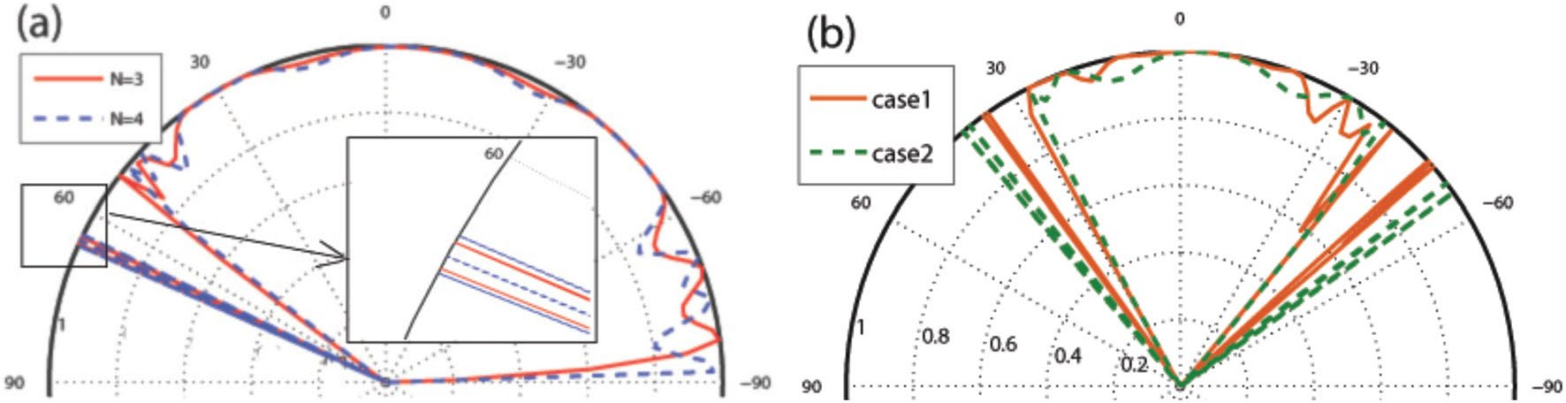
Angular profile of the total sideband transmission with $V_0=10$ and $V_1=0$ for (**a**) both configurations with $E=5$ and $N=3, 4$ with the zoomed resonances and (**b**) $N=3$ and $E=6$ for the latter and former profiles. Other parameters are the same as in Fig. 3.

**Figure 16:**
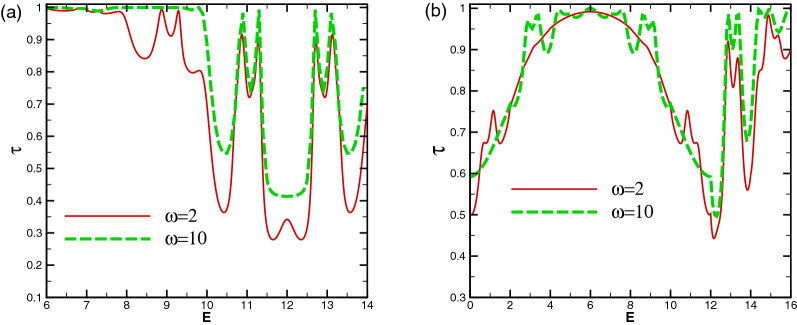
The total normal sideband transmission as a function of the energy for (**a**) the former and (**b**) the latter profiles with $N=3$, $V_0=12$ and $V_1=1.98$ for $\omega =2, 10$. Other parameters are the same as in Fig. 3.

The resonance splitting angle spaces are shown in Fig. 15 for different values of energy and number of the magnetic blocks with $V_0=10$ in the former and latter profiles. In the angular interval given by $\vert \sin \varphi +A/E\vert <\vert 1-V_0(x)/E\vert$, for both configurations, the perfect transmission is observed due to the propagating states inside the blocks. It experiences some fluctuations because of the Fabry-P$\acute{e}$rot resonances and the Klein scattering induced by the electrostatic barrier. It yields $-90^\circ<\varphi <53.1^\circ$ for $E=5$ and $-41.8^\circ<\varphi <30^\circ$ for $E=6$. For the special value of $E=V_0/2$ for the energy, the angular profiles of the former and latter profiles coincide (see Fig. 15a), while they are separated for other values of the energy, as shown in Fig. 15b. The $(N-1)$-fold resonances are observed out of the angular interval of the propagating state’s lobe. It has been observed that, by applying $V_1$, some fluctuation peaks are created with non-perfect transmission. The presence and absence of the oscillating scalar potential can be used as a characteristic of the transport properties of the studied magnetic systems. In the whole figures, up to now, the frequency has been set on $\omega =2$. Changing $\omega$ does not affect the whole discussed physical results. In Fig. 16, the normal transmission has been plotted for $\omega =2, 10$ in both configurations. The $(N-1)$-fold peaks are observed for both frequencies. To avoid the divergency due to the oscillatory nature of the Bessel functions discussed before, it should be noted that $\omega >V_1/n$.

### Conductance

**Figure 17:**
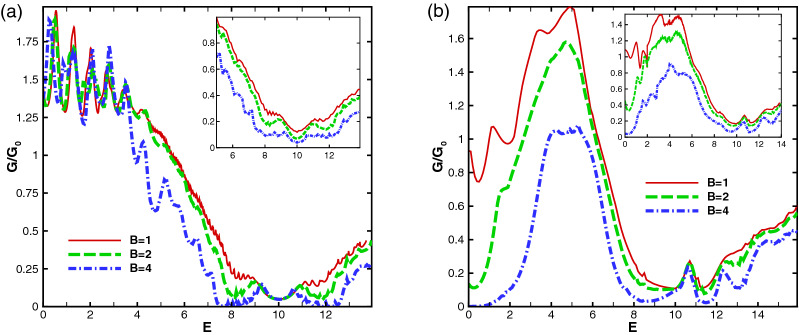
The total sideband conductance as a function of the energy for (**a**) the former and (**b**) the latter configurations with $N=2$, $V_0=10$, $V_1=0$ and different values of the magnetic field strength. The insets are sketched for $V_1=1.98$. Other parameters are the same as in Fig. 3.

The effects discussed up to now related to the transmission is, also, reflected in the total sideband conductance of the studied magnetic system. In Fig. 17 the total sideband conductance of the system is plotted versus the energy for $N=2$, $V_0=10$, $V_1=0$ and different value of *B* for the former (Fig. 17a) and latter (Fig. 17b) configurations, respectively. In the first profile, the conductance experiences a non-zero minimum in $E=V_0$ like the normal transmission. This minimum conductance approaches to zero by increasing the number of blocks up to $N\sim 6$ in the absence of $V_1$ while this happens, in the presence of $V_1$, for $N\sim 40$. In the second configuration, as is shown in Fig. 17b, the conductance has two minimums around $E=0$ and $E=V_0$ which are attributed to the well and barrier modes and the second one has the same behaviors mentioned for the first configuration. The conductance experiences a resonance peak after $E=V_0$. The conductance reaches to a maximum around $E=V_0/2$ which decreases in the presence of $V_1$ and by increasing *B*. The conductance decreases by increasing *B* but there is a region around $E=V_0$ and after $E=V_0$ in the former and latter configurations, respectively, where the conductance is almost insensitive to the increment of the magnetic field. Applying $V_1$, removes the insensitivity of the conductance, as is shown in the insets in Fig. 17. The oscillations of the conductance, particularly shown in Fig. 17a, have already been observed in a similar experimental work^45^.

**Figure 18:**
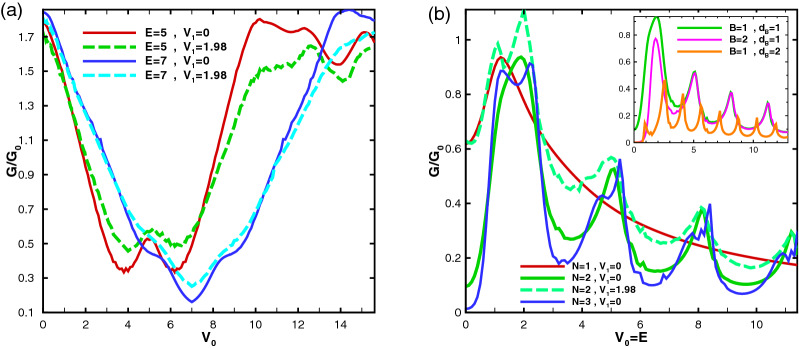
The conductance for the alternative static scalar potential profile as a function of the static scalar potential for (**a**) $N=2$ and different values of *E* and $V_1$ and (**b**) equal-barrier case varying *N* and $V_1$. The inset is for $N=2$ and different values of *B* and $d_B$. Other parameters are the same as in Fig. 3.

Figure 18 shows the dependence of the conductance to the static scalar potential for the alternative static scalar potential profile. As it is clear in Fig. 18a, the conductance comes to a minimum in $V_0=E$ except for some special values for the energy with a period depending on $d_B$. For $E=2, 5, 8, 11, \cdots$, the conductance reaches to a maximum at $V_0=E$. Switching $V_1$ on, the conductance increases around $V_0=E$ and decreases out of this region. In Fig. 18b the behavior of the conductance is studied for the equal-barrier case, i. e. $V_0=E$. For a single block structure, the conductance reaches uniformly to a maximum in low energies and then reduces to zero. For multiple blocks, the conductance has an oscillatory behavior with damping amplitude and a period depending on $d_B$. Increasing *N*, creates peaks in the maxima which was seen in Fig. 13 for normal transmission. The conductance increases by introducing $V_1$ for all values of $V_0=E$. In the inset of Fig. 18b, the effect of *B* and $d_B$ is studied for equal-barrier case. Increasing *B* does not affect the position of the peaks unless the amplitude of the first maximum is reduced but increasing $d_B$ reduces the amplitude and period of the conductance oscillations. In spite of the damping behavior of the transmission and conductance in the equal-barrier case in the first configuration and other perviously studied researches^16^, they have oscillatory behaviors in the second profile of the present work. This increasing behavior can be helpful in controlling and interrupting the transport.

**Figure 19:**
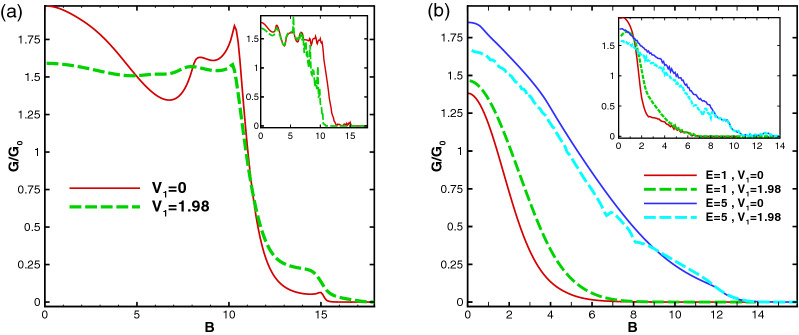
The sideband conductance as a function of the magnetic field’s strength for $N=1$, $E=1$ and $V_0=12$ for (**a**) former configuration and different values of $V_1$, and (**b**) latter profile for different values of *E* and $V_1$. The insets are sketched for $N=6$. Other parameters are the same as in Fig. 3.

Figure 19 illustrates the conductance as a function of the strength of the magnetic field for the first (Fig. 19a) and second (Fig. 19b) configurations, respectively. In accordance with the behavior of normal transmission, studied through Fig. 14, the conductance is suppressed after the second critical magnetic field $B_{c2}$ for both profiles. Its magnitude is about 1.55 T and 0.69 T by consideration of $B_0=0.1$ T for $N=1$, $E=1$ and $V_1=0$ for the former and latter configurations, respectively. It decreases by increment of *N*, as is shown in the insets of Fig. 19. The critical field $B_{c2}$ for the latter profile is less than the case for the former configuration with the same parameters. In spite of the fact that the normal transmission is unity for weak magnetic fields ($B<B_{c1}$) in the first configuration, the conductance decreases by applying $V_1$ which can be useful in designing of graphene-based nanostructures. The oscillations in the insets are associated with the Fabry-P$\acute{e}$rot fringes.

## Conclusion

In summary, we have studied the transport properties of a graphene sheet subjected to the magnetic blocks (barriers and wells) with the time-dependent scalar potential barriers. Two configurations of uniform and alternative static scalar potential have been investigated through the magnetic barriers and wells. It has been shown that the non-zero magnetic flux can confine DW quasiparticles in both configurations and the presence of the oscillating scalar potential,$V_1$, suppresses this confinement. We concentrated on the states with zero magnetic flux. The wave vector filtering effect has been observed for DW quasiparticles, in the absence of $V_1$ for the equal-barrier case (with the energy equal to the static scalar potential, i.e. $E=V_0$) which is enhanced by increasing *E* and the number of magnetic blocks, *N*. The transmission is suppressed over increasing the width of the magnetic blocks, in both profiles, and the critical width is associated with the cyclotron orbit diameter for DW fermions in the presence of the magnetic field. The normal transmission and the conductance vanishes, drastically, for both configurations in equal-barrier case by increasing *N*, *B* and decreasing $V_1$. The resonance effects have been observed specially in the former configuration. The magnetic system in the first configuration becomes full transparent in a wide angular range with the suitable choice for the parameters. The $(N-1)$-fold resonance splittings, also, have been shown for normal transmission around the equal barrier energy, which satisfies the non-degenerate eigenlevels and the region arising from the investigated model. So, the transport properties of the magnetic graphene system creates a reduction region by adjusting *E* and $V_0$. Out of this region in the first configuration, the Klein tunneling occurs but in the second one it is observed in a finite region around $E=V_0/2$. The $(N-1)$-fold resonances have been observed in the transmission and correspondingly some peaks in the conductance, for special values of $E=V_0$, in the second configuration which their distances depends on the width of the magnetic blocks. These $(N-1)$-fold resonances are, also, observed by variation of the strength of the magnetic field and the angle of incidence in both configurations. An estimation has been found for the place of this transmission reduction region. It has been shown that it is possible to switch the transport properties of the system by changing the system’s characteristic parameters as $V_1$, *B* and *N*. The phenomena studied here as realistic models are important from experimental point of view and we hope that our results can be useful in designment of graphene based electronic devices.
